# Case Report: Treatment for steroid-refractory immune-related myocarditis with tofacitinib

**DOI:** 10.3389/fimmu.2022.944013

**Published:** 2022-09-15

**Authors:** Qian Xing, Zhongwei Zhang, Biao Zhu, Qionghua Lin, Lihua Shen, Fangfang Li, Zhili Xia, Zhiyong Zhao

**Affiliations:** ^1^ Department of Critical Care, Fudan University Shanghai Cancer Center, Shanghai, China; ^2^ Department of Oncology, Shanghai Medical College, Fudan University, Shanghai, China

**Keywords:** immune checkpoint inhibitors, myocarditis, steroid-refractory, immunosuppression, tofacitinib

## Abstract

**Introduction:**

Immune therapy has ushered in a new era of tumor treatment, at the expense of immune-related adverse events, including rare but fatal adverse cardiovascular events, such as myocarditis. Steroids remain the cornerstone of therapy for immune-related myocarditis, with no clear consensus on additional immunosuppressive treatment for steroid-refractory cases yet.

**Case report:**

Here, we report a patient with stage IV nasopharyngeal carcinoma who developed immune-related myocarditis in the fourth course of therapy with immune checkpoint inhibitors. The patient presented with precordial discomfort with elevation of cardiac enzymes and interleukin-6, atypical electrocardiographic abnormalities, and reduced left ventricular ejection fraction. Coronary computed tomography angiography excluded the possibility of acute coronary syndrome. The therapy with tofacitinib targeting the Janus kinase-signal transducer and activator of transcription signal pathway was successfully conducted, since there was no significant improvement in troponin under high-dose steroid and intravenous immunoglobulin treatment. The patient recovered without major adverse cardiac events during hospitalization.

**Discussion:**

The safety and efficacy of tofacitinib in a patient with steroid-refractory immune-related myocarditis were investigated, hoping to provide a basis for prospective therapeutic strategies. Tofacitinib led to remarkable remissions in primary autoimmune disease by blocking the inflammatory cascade, indicating its potential therapeutic use in immune-related adverse events.

## Introduction

Immune checkpoint inhibitors (ICIs) potentiate T cell cytotoxicity against tumor cells through targeting programmed cell death 1 (PD-1), programmed cell death-ligand 1, or cytotoxic T-lymphocyte-associated protein 4, preventing the escape of tumor cells from the immune system. ICIs have revolutionized cancer treatment over the past decade, although at the expense of immune-related adverse events (irAEs), which are clinically detected in 70–90% of patients on ICIs (1). Meanwhile, high grades of irAEs are detected in 10–15% of patients, among which, cardiovascular adverse events could manifest as arrhythmias, pericardial disease, vasculitis, Takotsubo-like syndrome, dyslipidemia and, most frequently, myocarditis with a fatality rate over 50% (1–4). Here, we review the treatment of a patient with nasopharyngeal carcinoma, who presented with precordial discomfort with an elevation of cardiac enzymes who was hospitalized with immune-related myocarditis and treated with tofacitinib. We hope that this report provides a basis for prospective therapeutic strategies for a new option for treatment with tofacitinib, in the absence of clinical trials, and to improve the prognosis of immune-related myocarditis. Written informed consent was obtained from the patient in this report.

## Case presentation

A 67-year-old male was admitted to hospital in January 2021 with a new onset mass in the right neck. Magnetic resonance imaging indicated a thickening and enhancement of the nasopharyngeal wall, as well as multiple cervical lymphadenopathies. A nasopharyngoscopy revealed a neoplasm on the top left wall of the nasopharynx. Pathological findings of the mass confirmed non-keratinizing squamous cell carcinoma. Multiple bone metastases were detected by radionuclide bone scans. As a result, he was diagnosed with nasopharyngeal carcinoma (cT4N3M1).

He then participated in a clinical trial for metastatic nasopharyngeal carcinoma at initial diagnosis investigating the efficacy and safety of a PD-1 inhibitor (toripalimab) combined with gemcitabine and cisplatin (toripalimab 240 mg on day 1 + gemcitabine 1 g/m^2^ on days 1 and 8 + cisplatin 50 mg on days 1–3). There were no adverse reactions in the first three courses of treatment. However, he complained of precordial discomfort on the sixth day of the fourth course, in the absence of any history or heart disease. Blood results showed increased high-sensitivity cardiac troponin (hs-cTn) up to 0.553 (normal 0.013–0.025) ng/ml, creatine kinase-MB (CK-MB) up to 176 (normal 0–2.88) ng/ml, serum myoglobin up to 405 (normal 28–72) ng/ml, pro-brain natriuretic peptide (proBNP) up to 205 (normal 0–14.8) pmol/L, and interleukin-6 (IL-6) up to 17.41 (normal 0–5.4) pg/ml. While the value of IL-10 and interferon (IFN)–γ were, respectively, 17.2 and 26 pg/ml, slightly higher than the normal range.

He was then transferred to ICU. QT prolongation, horizontal ST segment depression (0.5–1 mm) of V5 and V6 leads, and T wave inversion in I, AVL, II, III, AVF, V5, and V6 leads were observed following a dynamic electrocardiogram. A bedside echocardiogram suggested the left ventricular ejection fraction (LVEF) decreased to 40%. A coronary computed tomography angiography showed that the proximal occlusion in left anterior descending, right coronary artery, and left circumflex branch was 70, 90, and 90%, respectively, with collateral formation and distal development, indicating the diagnosis of chronic and already compensatory coronary heart disease. Therefore, the new onset of chest symptoms and laboratory abnormalities were inferred as immune-related myocarditis and the patient was excluded from the clinical trial. He was quite depressed about it.

We immediately continued immunosuppressive therapy with methylprednisolone 4 mg/kg per day intravenously, as well as anti-platelet therapy with aspirin and anti-atherosclerosis therapy with atorvastatin. However, there was no improvement with his clinical performance; indeed, we noted an aggravation in the value of the myocardial zymogram. Consequently, intravenous immunoglobulin (IVIG, 0.4g/kg per day for 5 days) and a higher dosage of methylprednisolone (500 mg for 3 days, followed by a step-by-step dose reduction) were administered on the second day in the ICU, combined with trimetazidine and coenzyme Q10 to nourish cardiac muscle. There was a subsequent decline in the value of the myocardial zymogram as his symptoms improved.

**Figure 1:**
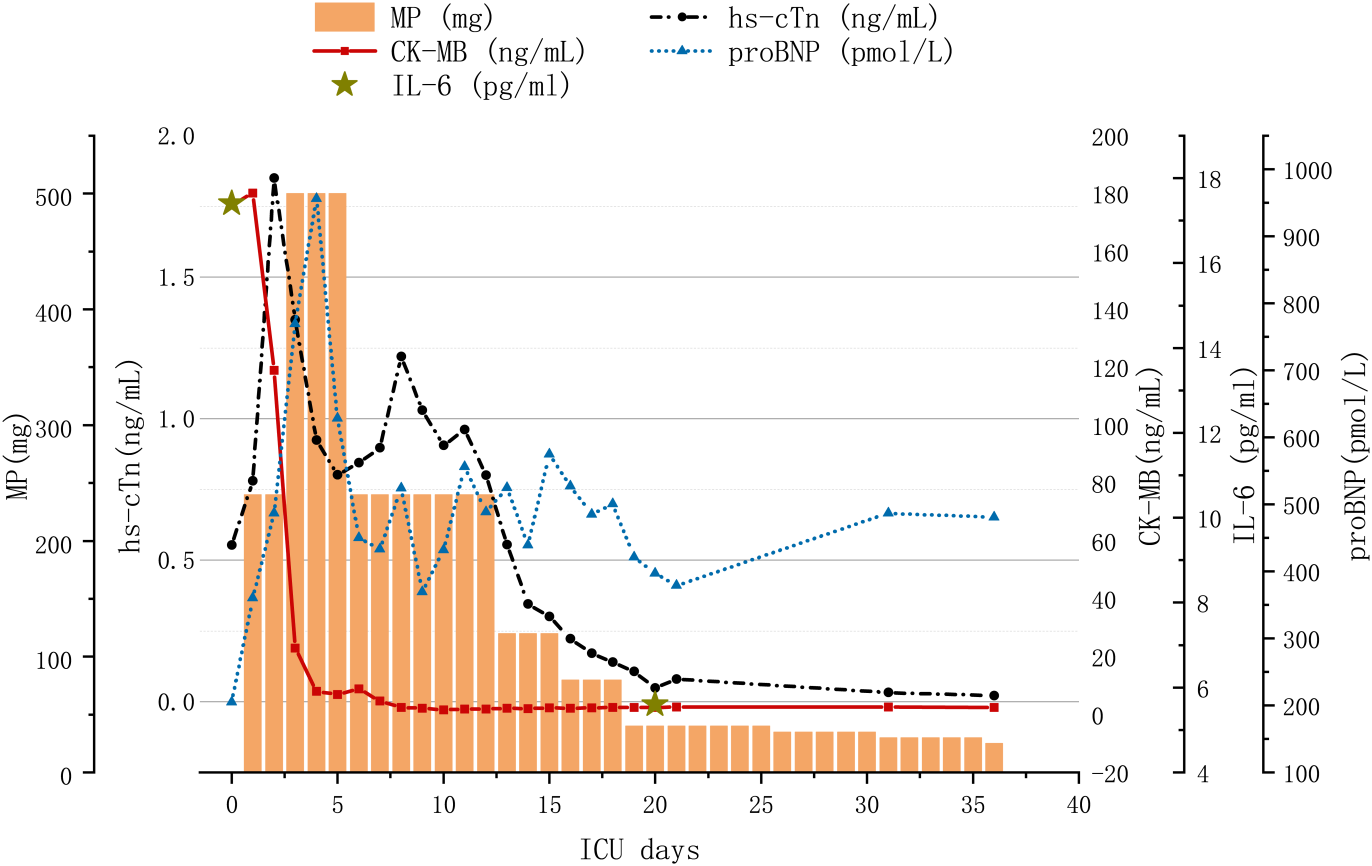
Variation in lab tests and treatment strategy with immune-related myocarditis. MP, methylprednisolone; hs-cTN, high-sensitivity cardiac troponin; CK-MB, creatine kinase MB Form; proBNP, pro-brain natriuretic peptide; IL-6, interleukin-6; IVIG, intravenous immunoglobulin.

However, this was short lived; the value of hs-cTn elevated on the sixth day since admission to ICU. Considering the aggravated laboratory parameters, he was treated with tofacitinib (5 mg twice daily for a week) for intensive immunosuppression. The level of hs-cTn, CK-MB, IL-6, IL-10, and IFN-γ gradually decreased to nearly normal a month after admission. Variation in lab tests and treatment strategies are demonstrated in **Figure 1**. Regular cardiac assessment by electrocardiogram and echocardiogram showed few changes over time. The patient felt much better recovering without a major adverse cardiac event (MACE), and his confidence to fight the tumor was restored. Unfortunately, the enlarging neck mass indicated tumor progression and he was transferred to the oncology department for further anti-tumor therapy.

## Discussion

Immune-related myocarditis often occurs in men (71%); studies show that its prevalence was only 1.14% with a mean onset age of 65 ± 13 years and a median onset time of 34 days after the initiation of ICIs (3). The ensuing myocarditis, 3 months later, exhibited significantly worse heart failure with left ventricular systolic dysfunction compared to the relatively early cases happening within the first three months (5). Consistent data suggest that patients receiving combination ICIs or with pre-existing cardiovascular disease had an increased incidence of myocarditis, whereas diabetes mellitus and obesity were unclear as risk factors (1). Patients with immune-related myocarditis could present from asymptomatic to acute respiratory or cardiac syndrome; 46% of the cases experienced MACE, including ventricular arrythmias, complete heart block, cardiogenic shock, cardiac arrest, and cardiovascular death, leading to high mortality (6, 7). In addition, clinicians should be alerted that complicated neuromuscular irAEs could occur with immune-related myocarditis, since there are increasing reports of the triple M overlap syndrome of myositis-myocarditis-myasthenia gravis (8–10). Consequently, a prompt and accurate diagnosis is critical for initiating treatment.

It is reported that nearly all cases with myocarditis demonstrated an elevated troponin (94%) and abnormal electrocardiogram (89%), whereas 66% had an increased proBNP and 51% presented with preserved LVEF on their echocardiograms (3). Higher troponin, CK, and CK-MB levels were associated with increased mortality, whereas the presence of prolonged QRS duration trended toward increased the risk of subsequent MACE (11, 12). Compared with LVEF, which was also measured by echocardiogram, a lower global longitudinal strain was strongly associated with the incidence of MACE in myocarditis patients (13, 14). Retrospective studies suggested that the application of T1 mapping with cardiac magnetic resonance can provide diagnostic value of immune-related myocarditis and predict the risk of MACE (15, 16). In addition, 68 Ga-FAPI PET/CT, reduced absolute lymphocyte count and increased neutrophil/lymphocyte ratio were also identified as new methods to detect immune-related myocarditis (17, 18). As the gold-standard test, endomyocardial biopsy indicated infiltration with T lymphocytes and macrophages among immune-related myocarditis cases, although rarely pursued for its invasive nature and potential complications (19, 20). In addition, the application of novel techniques, such as single-cell RNA sequencing and the gene knockout mice model, may allow better characterization of infiltrating immune cells (21, 22). On the basis of these tests, this patient was definitively diagnosed with myocarditis under the criteria by Bonaca (23).

The precise mechanisms of immune-related myocarditis still remain unclear. Suggested possibilities include the inhibition of the cardio-protective PD-1 and PD-L1 pathways by hyperactivated cytotoxic T cells in myocardiocytes, shared antigens between the tumor and the myocardium (24). Prompt initiation of steroids are recommended as first line therapy by the latest global guidelines, lacking consensus on the initial dosing or tapering protocols (25). Intensified immunosuppressive therapy (IIST), including abatacept, mycophenolate mofetil, IVIG, alemtuzumab, infliximab, and anti-thymocyte globulin, should be added as soon as evolution is unfavorable within 24 h on steroids especially when hemodynamic/electrical instability or neuromuscular adverse events occur (25–27). However, recommendations from current guidelines for IIST were adapted from the experience of primary autoimmune diseases, without taking into consideration the personalized treatments under specific immunohistopathological findings of affected organs (28).

**Figure 2:**
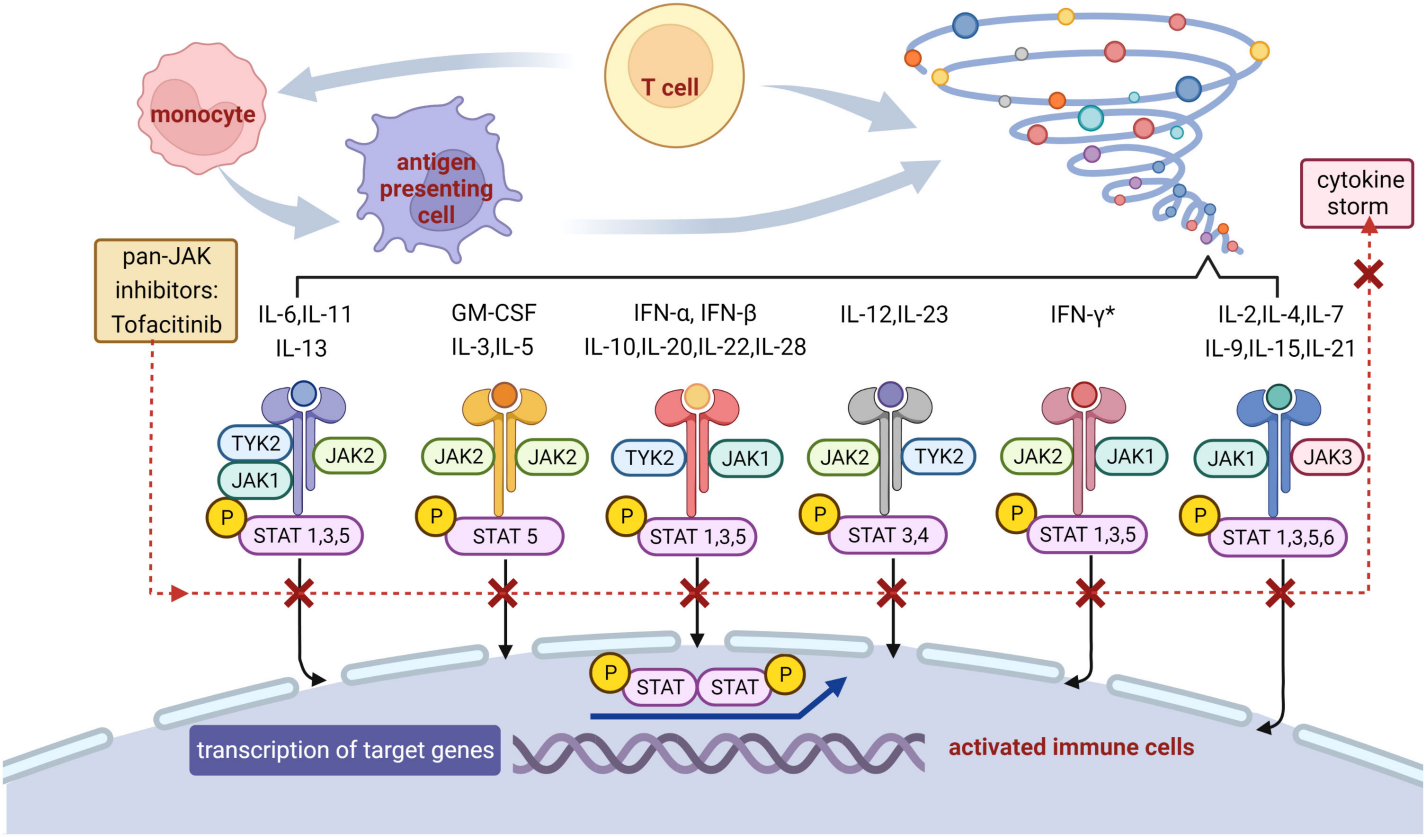
Potential mechanism of the blockade on inflammatory storm with tofacitinib. JAKs phosphorylate tyrosine residues on their cytokine receptors, leading to recruitment and phosphorylation of STATs. Phosphorylated STATs dimerize and translocate to the nucleus, where they promote the transcription of genes encoding proteins with functions in proliferation and inflammation. Tofacitinib binds to JAKs thus prevents downstream gene transcription, resulting in reduction in cytokine storm and inflammatory cascade. * Tofacitinib has showed efficacy for immune-related colitis, probably through the inhibition of IFN-γ signaling. JAK, Janus kinase; STAT, signal transducer and activator of transcription; P, phosphorylation; TYK, tyrosine kinase; IL, interleukin; GM-CSF, granulocyte macrophage colony stimulating factor; IFN, interferon.

Except for the IIST recommended by current guidelines, Janus kinase (JAK) inhibitors and IL-6 receptor inhibitors have attracted much attention with their performance on irAEs. As the first generation of pan-JAK inhibitors, tofacitinib primarily suppressed JAK1/JAK3, disturbed the JAK–signal transducer and activator of transcription (STAT) pathway downstream of inflammatory cytokine receptors, and blocked most inflammatory cytokines, including IL-6 and the subsequent inflammatory storm (**Figure 2**) (29–31). IL-6, a critical driver of inflammation, can also induce the differentiation of pathogenic TH17 from naive T cells and inhibit the regulatory T cells, in addition to the JAK-STAT pathway (32). Tofacitinib has been approved for rheumatoid arthritis, psoriatic arthritis, and ulcerative colitis and was recommended for immune-related colitis (33). The differentiation from CD8^+^ tissue-resident memory T cells to cytotoxic T-lymphocytes-induced cytokines, including IFN-γ, was considered to participate in the emergence of immune-related colitis (34). Tofacitinib has showed efficacy for immune-related colitis, probably through the inhibition of IFN-γ signaling (**Figure 2**) (33, 35). The patient in our case presented with elevation of IL-6, IL-10, IFN-γ, and deterioration in troponin under therapy with steroids and IVIG, was treated with tofacitinib to target JAK-STAT signal pathway and inhibit these cytokines. In addition, highly expressed phosphorylated proteins with the JAK-STAT pathway were found in the rat model of autoimmune myocarditis, suggesting that the JAK inhibitors may suppress the development of myocarditis (36).

Tofacitinib has been reported to be beneficial to treat steroid and cyclosporin refractory myocarditis result from drug rash with eosinophilia and systemic symptoms by inhibition of cytokines with an anti-eosinophilic effect (37). Wang et al. proposed a new classification of steroid-resistant myocarditis based on troponin rebound during corticosteroid tapering, and tofacitinib was proven clinically beneficial for these patients (38, 39). Tocilizumab, a humanized monoclonal antibody for IL-6, was reported to behave possible synergistic anticancer effect with ICIs and efficacy against irAEs including myocarditis (40–42). However, similar to tofacitinib, only limited cases could support the efficacy for myocarditis, lacking evidence-based medical proof. More recently, a multicenter randomized placebo-controlled trial among patients hospitalized with COVID-19 pneumonia demonstrated that tofacitinib led to a lower risk of death and respiratory failure (43). The action of tofacitinib on multiple critical pathways of the inflammatory cascade may ameliorate inflammation-driven lung injury caused by COVID-19 (43). Considering similar immune dysfunction and potential cytokine storms in immune-related myocarditis, tofacitinib holds promise owing to its oral route of administration and its ability to disrupt both innate and adaptive branches of the immune system (28). Although loss-of-function mutations in JAK are worried to be associated with subsequent inefficacy of immune therapy, most of our patients indeed had to discontinue their immunotherapy permanently once diagnosed with immune-related myocarditis (32, 44). The long-term safety associated with tofacitinib in RA exhibited risks of MACE, which will not be underestimated if used in the setting of immune-related myocarditis (44).

In conclusion, tofacitinib can offer additional clinical benefit in patients with steroid refractory and provides a new option for the IIST strategy in immune-related myocarditis. In the future, further meta-analysis based on more cases and large-scale trials may be conducted to explore the drug safety, efficacy, and intensive mechanism of tofacitinib. With the variable manifestation and various immunosuppressive agents, it is essential to involve a multi-disciplinary team to optimize management of these complications in patients.

## Data availability statement

The raw data supporting the conclusions of this article will be made available by the authors, without undue reservation.

## Ethics statement

Written informed consent was obtained from the individual(s) for the publication of any potentially identifiable images or data included in this article.

## Author contributions

(I) Conception and design: QX, BZ. (II) Administrative support: QL, LS. (III) Provision of study materials or patients: FL. (IV) Collection and assembly of data: QX, ZX, ZYZ. (V) Data analysis and interpretation: QX, ZWZ. (VI) Manuscript writing: QX. All authors contributed to the article and approved the submitted version.

## Acknowledgments

The authors thank the patient and his family for allowing the publication of this work.

## Conflict of interest

The authors declare that the research was conducted in the absence of any commercial or financial relationships that could be construed as a potential conflict of interest.

## Publisher’s note

All claims expressed in this article are solely those of the authors and do not necessarily represent those of their affiliated organizations, or those of the publisher, the editors and the reviewers. Any product that may be evaluated in this article, or claim that may be made by its manufacturer, is not guaranteed or endorsed by the publisher.
